# Tuberculous cold abscess of the chest wall: A clinical and surgical experience. Report of 16 cases(Case series)

**DOI:** 10.1016/j.amsu.2020.02.001

**Published:** 2020-02-13

**Authors:** El Hassane Kabiri, Essotina Ayouba Alassane, Maruis Kemini Kamdem, Mohamed Bhairis, Mouad Amraoui, Faycal El Oueriachi, Massine El Hammoumi

**Affiliations:** a Department of Thoracic Surgery, Mohamed V Military Teaching Hospital, Morocco; b Faculty of medicine -Mohamed V University, Rabat, Morocco

**Keywords:** Tuberculosis-cold abscess-chest wall, Debridement

## Abstract

**Background:**

Tuberculosis is a public health problem in developing countries. Tuberculosis of the chest wall is rare and often presents as cold abscess (to differentiate from pyogenic abscess) or pseudotumoral mass whose diagnosis is difficult and often requires a surgical biopsy.

**Patients and methods:**

The medical series of 16 patients with cold chest wall abscess treated with surgery in association to anti-tubercular therapy were analysed retrospectively for the period of 7 years between January 2011 to December 2017 at Mohamed V Military Teaching Hospital – Rabat - Morocco.

**Results:**

The clinical examination provided a correct preoperative diagnosis of the abscess in all cases. Five patients had a past history of pulmonary tuberculosis and three patients had concomitant active infection. There were 6 cases on the left side, 9 cases on the right side and one case on the anterior chest wall. All patients underwent surgical drainage and debridement with specimens for bacteriology and histology. It was not necessary to resect ribs or sternum in all cases (sample costal or sternal curettage in one case each). Anti-tubercular treatment was routinely administered (6–9 months) with drug combinations of Isoniaside, Rifampicin, Pyrazinamide and Ethambutol. The evolution was favorable in all cases without complications or recurrences.

**Conclusion:**

Drainage of chest wall abscess and complete debridement provide adequate treatment. Post-operative anti-bacillary therapy should be combined with surgical procedures to minimize local complications and recurrence of infection.

## Highlights

• Tuberculous abscesses of the chest wall well be associated pulmonary disease or primary location.
• Chest CT scan is the best exam to diagnosis and the procedure to do.
• Debridement must be large and in association with antituberculosis chemotherapy.

## 1 Introduction

**Fig. 1:**
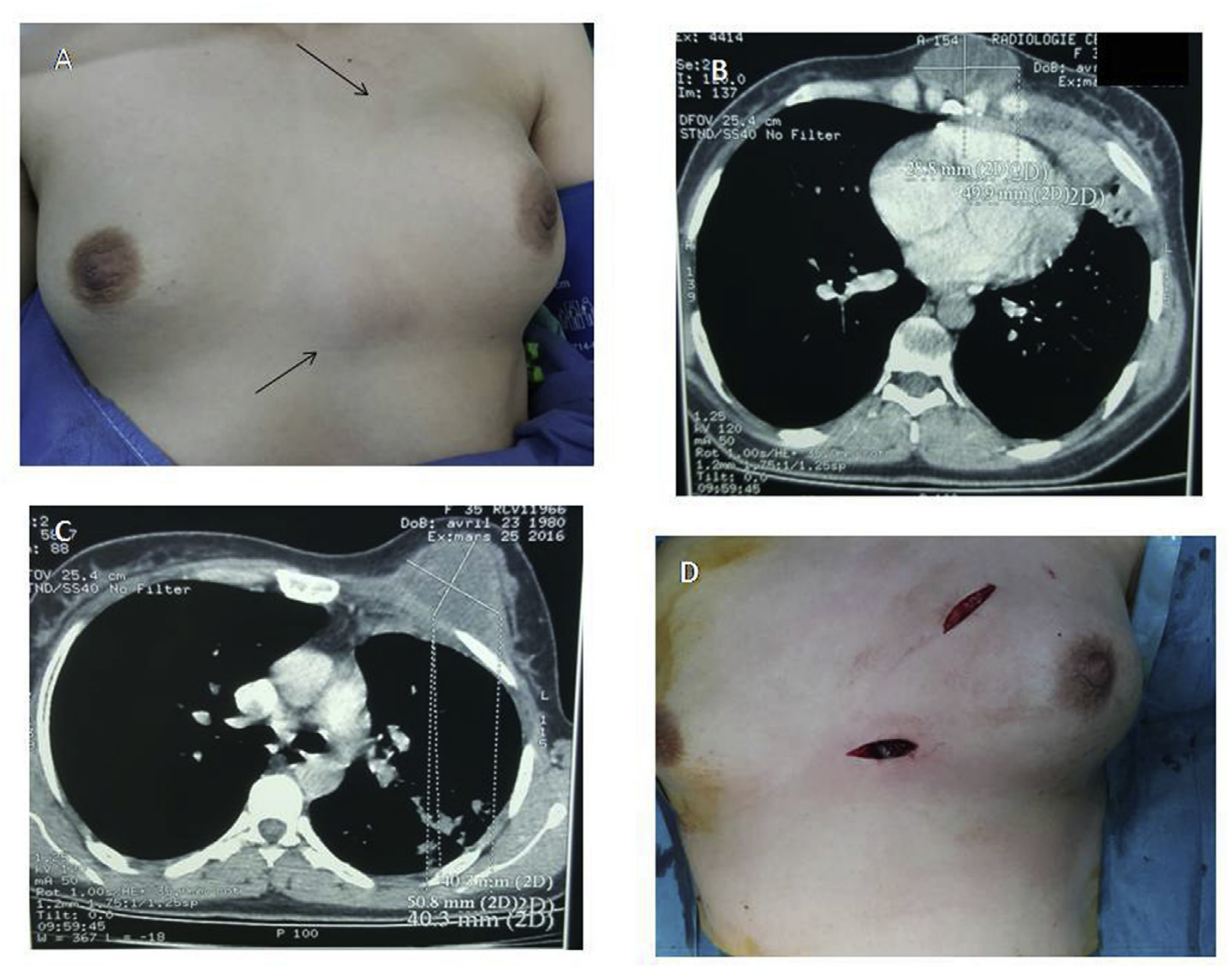
A: Two swelling anterior chest wall in the left side just up to the breast and the second next to the xyphoid appendix. B and C: Chest computed tomography showing two anterior abscesses (median and left side). D: Double incision for drainage the collections and wide debridement (Patient no: 9).

**Fig. 2:**
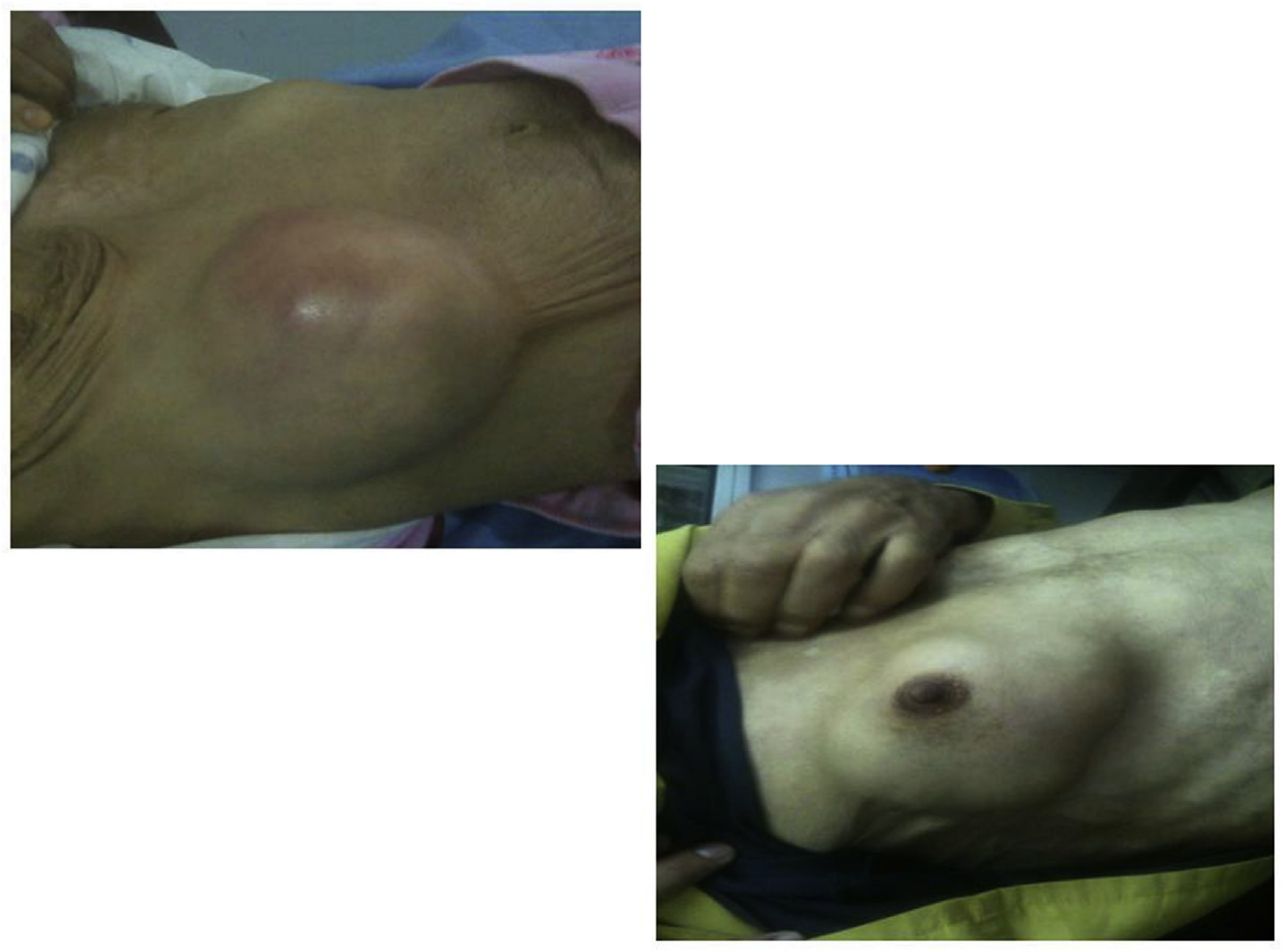
Voluminous fluctuant anterior-lateral chest wall swelling (Patient 5 and 8).

**Fig. 3:**
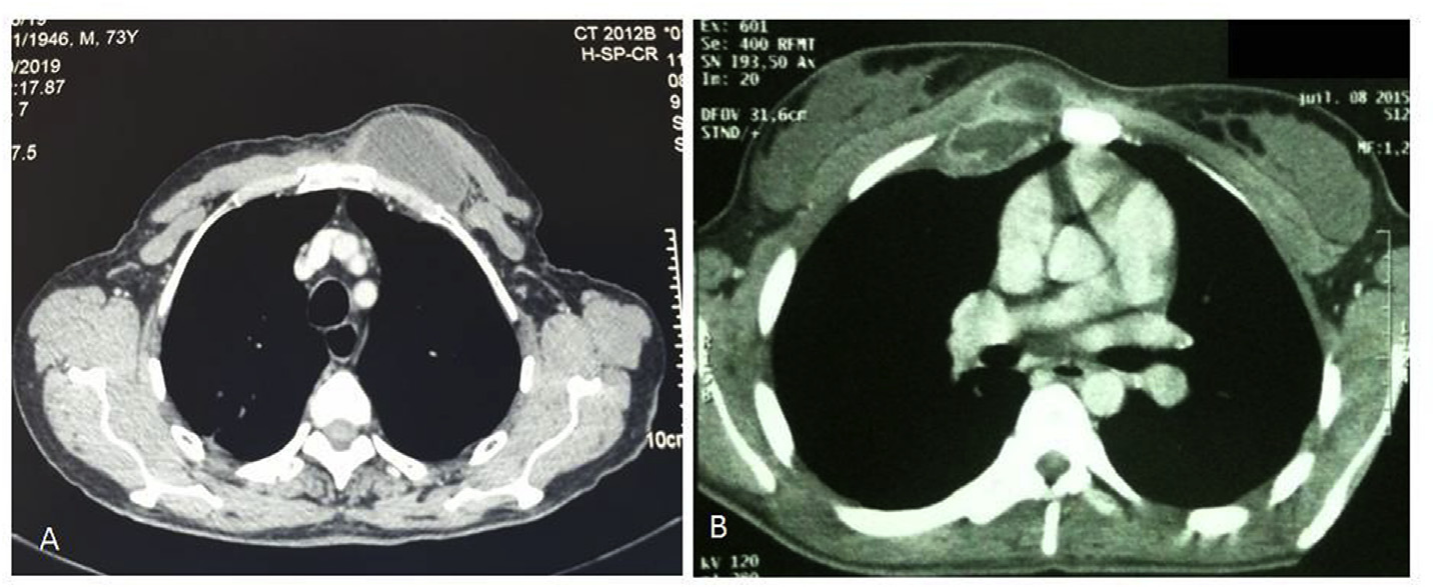
A: Chest computed tomography showing anterior chest wall mass (Patient *no: 16*) B: Chest computed tomography findings showing anterior chest wall abscess with pleural involvement and multilocular form. *(Patient no: 12)*.

Tuberculosis (TB) is a public health problem in developing countries. It's still relevant in Morocco. The thoracic wall localization is exceptional and presents a diagnostic problem, particularly with chest wall tumors and other pyogenic wall infections and actinomycetes infections. It is very often the sign of severe and disseminated tuberculosis [1,2] and may be isolated or secondary to pleuropulmonary involvement [3] (see Table 1, Fig. 1, Fig. 2, Fig. 3).

The diagnosis of tuberculosis of the chest wall is suspected by clinical examination including general or pleuropulmonary symptoms, radiographic signs, and confirmed by bacteriology and/or histology data. Therapeutic management combines medical treatment with surgical excision or drainage.

We report our experience of 16 patients immunocompetent with chest wall tuberculosis but spinal (Pott's disease) and breast tuberculosis were excluded.

## 2 Patients and methods

This is a retrospective study of 16 cases of cold TB abscess collected at the thoracic surgery department of the Mohamed V Military Teaching Hospital in Rabat - Morocco. All patients with empyema or abscess of the chest wall without histological or bacteriological confirmation were excluded from the study. Thus 16 cases were selected, there were 12 men and 4 women aged 39.1 years on average (extremes: 18–73 years). The abscess was inaugural on the 11 cases and only 5 patients had a history of tuberculosis dating back respectively to 6 years and 10 years. No other site of active tuberculosis was noted, the symptomatology was mainly dominated by chest pain and swelling chest wall (Fig. 1, Fig. 2 A, 2).

All patients had chest x-ray and thoracic CT scans (Fig. 1, Fig. 3). Costal lysis was observed in 2 patient and sternal lysis in 1 patient. All the patients retained were operated in the same day of admission to the emergency department, ten under general anesthesia and six under local anesthesia associated with sedation. They all benefited a flattening of the abscess by elective incisions (Fig. 1D), associated with resection of necrotic tissue. Sternal or costal curettage was performed in two patients. Samples of pus, necrotic tissue and bone were sent in bacteriology for research of acid-fast bacilli (AFB) either directly or by culture on Lowenstein milieu or by GeneXpert study. Direct examination was negative in all patients, culture positive in 7 cases and GeneXpert positive in 14 patients. Postoperative follow-up was simple for all our patients. The pathological lesions characteristic of tuberculosis (giant cell granuloma and caseous necrosis) were identified on fragments sent to pathological anatomy. Thus, after a liver test, each patient received a tuberculous chemotherapy of 6–9 months in accordance with the protocol adopted in Morocco.

The average duration of hospitalization was 6,7 days. No recurrence, no deaths were observed in our patients after minimally two years of surveillance. All patients are asymptomatic and have returned to normal occupational and physical activity (Table 1).

**Table 1:** Summary of findings data in16 patients with chest wall tuberculosis.

Case	Age/sex	History of tuberculosis	Location	Clinical signs	CT scan	Operation	GeneXpert	Histology	Culture	Drugs	Outcome
1	35/M	None	Left	Chest pain	Chest mass	D	+	Caseous necrosis	+	2 RHZE 4 RH	Good
2	28/F	concomitant	Right	Palpable mass	-chest mass Parenchymal infiltration	D	+	Caseous necrosis	**-**	2RHZE 7RH	Good
3	18/M	None	Right	Palpable mass	Chest mass	D	+	Caseous necrosis	**-**	2RHZE 4 RH	Good
4	34/M	Past	Left	Palpable mass	Chest mass	D	+	Caseous necrosis	+	2 RHZE 4 RH	Good
5	45/F	None	Right	Palpable mass	Chest mass (11 × 8cm)	D	+	Caseous necrosis	**-**	2 RHZE 4 RH	Good
6	34 M	None	Left	Palpable mass	Chest mass (7,8 × 6,5cm)	D	+	Caseous necrosis	+	2 RHZE 4 RH	Good
7	38/M	Past	Right	Palpable mass	Chest mass	D	+	Caseous necrosis	**-**	2 RHZE 4 RH	Good
8	57/M	None	Right	swelling	Chest mass (7,3 × 5,5cm)	D	+	Caseous necrosis	**-**	2 RHZE 4 RH	Good
9	52/F	Past	Left/Median	Chest pain Palpable mass	Chest mass (5 × 3cm and4x5,1 cm) Sternal erosion	D + S	+	Caseous necrosis	+	2 RHZE 4 RH	Good
10	45/M	concomitant	Right	Chest pain	**-** Chest mass Parenchymal infiltration	D	+	Caseous necrosis	+	2 RHZE 7 RH	Good
11	40/M	None	Left	Chest pain Palpable mass	Chest mass	D	**-**	Caseous necrosis	**-**	2 RHZE 4 RH	Good
12	19/F	Past	Right	swelling	Chest mass 8 × 5cm Multilocular lesion Pleural infiltration	D	+	Caseous necrosis	**-**	2 RHZE 4 RH	Good
13	26/M	None	Right	Chest pain	Chest mass Erosion of margin of the 5th rib	D + R	+	Caseous necrosis	+	2 RHZE 4 RH	Good
14	42/M	Past	Left	Chest pain	Chest mass Pleural effusion	D	**-**	Caseous necrosis	**-**	2 RHZE 4 RH	Good
15	35/M	None	Right	Chest pain	Chest mass	D	+	Caseous necrosis	+	2 RHZE 4 RH	Good
16	73/M	None	Left	Palpable mass Fever	Chest mass 6,5 × 4cm	D	+	Caseous necrosis	**-**	2 RHZE 4 RH	Good

D: Debridement - RC: Rib Curettage – SC: Sternal Curettage R: Rifampicin H: Isoniazid Z: Pyrazinamide E: Ethambutol.

## 3 Discussion

Tuberculosis of the chest wall is an extra-pulmonary location and represents 1%–5% of all musculoskeletal tuberculosis [2,[4], [5], [6]]. The sternal primary tuberculosis disease accounts for approximately 0, 3% [1] and only 31 articles in Pubmed database were found [7] and Tuberculosis of the ribs represents 2% [2].

Tuberculosis of the chest wall can affect all anatomical structures. Usual clinical feature are painless cystic masses without skin inflammatory signs on the skin giving the appearance of a cold abscess or a solid tissue mass and can be sometimes mobile. However, it affects the ribs more than the sternum, clavicle and vertebrae [8]. Rarely intercostal spaces are affected without bone involvement during cold subcutaneous abscess.

The incidence of chest wall tuberculosis will not decreased in the future, because of the recrudescence of multidrug resistant forms and increase of immunocompromised patients [2,8]. There is no predilection of sex or age while chest wall cold abscess was exceptionally described in children [[8], [9], [10]].

Three mechanisms have been described to explain the pathogenesis of chest wall tuberculosis: contiguous extension of pulmonary or pleural involvement, haematogenous dissemination, direct transcutaneous inoculation or extension from adenitis of the chest wall [2,4,8,11]. This latter mechanism is predominant, especially in cold subcutaneous thoracic abscesses where bone involvement is most often secondary to adenitis and generally leads to endothoracic extension.

Skin fistulization is rare and is often observed in case of delayed treatment, which is not the case of crofuloderma, which represents the clinical form of cutaneous satellite cutaneous disease with nodal or primary osteoarticular focus [3]. This mainly concerns the anterior intercostal ganglia, hence the parasternal location of cold abscesses. Tuberculosis of the chest wall may be isolated or associated with pulmonary or mediastinal or even multifocal localization [2,9]. In a region with a high TB endemicity, particularly in developing countries, there are some signs of appeal that evoke the diagnosis, namely the signs of tuberculosis impregnation, a history of tuberculosis and parietal swelling gradually increasing in volume without major associated inflammatory signs. X-rays of the thorax may be normal at the beginning [2] or may reveal pleural effusion, pleural thickening, or parietal opacity [2,5,12]. Ultrasonography is particularly useful for specifying the echogenicity of the parietal tumefaction content observed on clinical examination [2]. Thoracic CT is the test of choice for the exploration of tuberculous lesions of the chest wall, specifying the nature and extent of these lesions. It can also reveal the presence of bone lysis, intrathoracic lymphadenopathy or even pleuropulmonary lesions. However, he can be mistaken for diagnosis by directing him to a tumor origin. Scintigraphy is particularly useful for detecting mute bone sites [2,3,13]. Imaging plays a very important role but it does not allow to establish a differential diagnosis with tumors of the chest wall.

Since the radioclinic diagram is not specific, some diagnosis must be considered like non tuberculosis abscess, benign or malignant necrosis tumors [8]; the diagnosis of certainty is essentially based on the detection of the koch bacillus in the puncture fluid and/or in the biopsy fragments in direct examination and especially after culture in a specific medium. Histology shows a specific granuloma with caseous necrosis. The sample can be obtained surgically or directly by needle aspiration [7]. Surgical biopsy is an interesting alternative for diagnostic and therapeutic purposes, especially in case of cold or localized mass abscess [8, 9]. The geneXpert study technique is nowadays a rapid and effective diagnostic tool for tuberculosis compared to microscopy, which has a low sensitivity and often a long culture [14]. This technique allows early detection of tuberculosis cases, thus enabling the establishment of treatment and stopping the chain of transmission. Similarly, it can detect resistance to rifampicin [3,14].

The treatment of cold abscess is the subject of much controversy due to the rarity of the disease. Thus, several questions remain unanswered regarding the duration of treatment, its usefulness and the modalities of surgical treatment. The strong suspicion of tuberculous origin generally imposes a medical treatment from the beginning pending the results of the culture [11]. For some authors, a well-conducted medical treatment, lasting from 9 to 12 months, can cure alone [9,12,15]. However, there are very few series with a limited number of patients and a high recurrence rate. In most series, the combination of surgery and antituberculosis treatment is recommended, preferably to reduce the risk of recurrence [9,10,15]. The surgical procedure is essentially based on the obliteration of the residual cavity after flattening of the cold abscess and on the removal of all the infected tissues, including the affected ribs or cartilage segments [9,12,15]. This minimizes complications and postoperative recurrences.

Some authors suggest that surgical drainage of the abscess should be considered only if the needle aspiration is not enough to resolve the problem [7] we think that aspiration alone is not sufficient because there is always multiloculated sites that only surgical xide debridement can ensure good outcome and prognosis.

Paik et al. report some complications like: bleeding, subcutaneous emphysema, pleural effusion, empyema and possibility of activation of pulmonary tuberculosis [9].

The prognosis is often favorable, although it depends on the time of diagnosis and the speed of treatment [9,10,13]. The average duration of treatment of chest wall tuberculosis is 6 months and can be extended from 9 to 12 months depending on clinical presentation, bacillary load and response to treatment. Clinical, biological and radiological surveillance should be systematic. It can be used to monitor the progression of the disease and the effectiveness of the treatment, as well as to investigate possible complications.

## 4 Conclusion

Tuberculosis of the chest wall is exceptional and may be in the form of a cold abscess or a pseudo-tumor mass. In the absence of pulmonary or extra-pulmonary lesions suggestive of tuberculosis, it is difficult to distinguish a cold tuberculous abscess from a tumor of the chest wall. The diagnosis of tuberculosis of the chest wall should be discussed before signs of clinical and radiological attractiveness, particularly in endemic areas. The prognosis is generally better on multidrug therapy with an average duration of 6 months associated with complete surgical resection of the abscess thus reducing complications and recurrences.

## Ethical approval

There is no ethical committee in our country (Not applicable for this manuscript).

## Sources of funding

There are no sources of funding.

## Author's contribution

EHK composed the manuscript, and all the remaining authors provided critical edits to the final draft. All authors read and approved the final manuscript.

## Research registration number

Researchregistry5242.

## Guarantor

The Guarantor is the one or more people who accept full responsibility for the work and/or the conduct of the study, had access to the data, and controlled the decision to publish.

EK El Hassane Kabiri.

## Availability of data and materials

The datasets used and/or analysed during the current study available from the corresponding author on reasonable request.

## Consent for publication

Not applicable.

## Provenance and peer review

Not commissioned, externally peer reviewed.

## Declaration of competing interest

The authors have not conflict of interest.
